# Human Guanylate-Binding Protein 1 Positively Regulates Japanese Encephalitis Virus Replication in an Interferon Gamma Primed Environment

**DOI:** 10.3389/fcimb.2022.832057

**Published:** 2022-05-19

**Authors:** Simran Chhabra, Kiran Bala Sharma, Manjula Kalia

**Affiliations:** Regional Centre for Biotechnology, NCR Biotech Science Cluster, Faridabad, India

**Keywords:** Japanese encephalitis (JE) virus, interferon stimulated genes, guanylate binding protein, STAT 1, interferon gamma (IFNγ), flavivirus

## Abstract

RNA virus infection triggers interferon (IFN) receptor signaling, leading to the activation of hundreds of interferon-stimulated genes (ISGs). Guanylate-binding proteins (GBPs) belong to one such IFN inducible subfamily of guanosine triphosphatases (GTPases) that have been reported to exert broad anti-microbial activity and regulate host defenses against several intracellular pathogens. Here, we investigated the role of human GBP1 (hGBP1) in Japanese encephalitis virus (JEV) infection of HeLa cells in both an IFNγ unprimed and primed environment. We observed enhanced expression of GBP1 both at transcript and protein levels upon JEV infection, and GBP1 association with the virus replication membranes. Depletion of hGBP1 through siRNA had no effect on JEV replication or virus induced cell death in the IFNγ unprimed environment. IFNγ stimulation provided robust protection against JEV infection. Knockdown of GBP1 in the primed environment upregulated expression and phosphorylation of signal transducer and activator of transcription 1 (STAT1) and significantly reduced JEV replication. Depletion of GBP1 in an IFNγ primed environment also inhibited virus replication in human neuroblastoma SH-SH5Y cells. Our data suggests that in the presence of IFNγ, GBP1 displays a proviral role by inhibiting innate immune responses to JEV infection.

## Introduction

Japanese Encephalitis Virus (JEV), a mosquito-borne flavivirus, is the leading global cause of viral encephalitis, which results in 68,000 cases with 13,600–20,400 deaths each year. The virus is endemic mainly in East and South-East Asian countries (Quan et al., 2020; Sharma et al., 2021). In India, epidemics occur every year where many children succumb to the disease (Kulkarni et al., 2018). Although vaccines are available for JEV, no antiviral drugs or therapies have been developed. A detailed understanding of the host-virus interaction is critical for the development of effective antivirals (Turtle and Solomon, 2018; Sharma et al., 2021b). While there are a few reports that have suggested the induction of various interferon-stimulated genes (ISGs) at both transcription and protein levels during JEV infection (Yang et al., 2011; Zhang et al., 2013; Chauhan et al., 2021; Sharma et al., 2021a), the finer details of the innate immune landscape during JEV infection remain largely unexplored.

Virus infection activates IFN and JAK-STAT signaling which ultimately results in the upregulation of ISGs. The Guanylate-binding proteins (GBPs) belong to the GTPase superfamily of ISGs. There are 7 GBPs in human that are involved in various cellular functions such as inhibition of cell spreading and proliferation, activation of inflammosome and antimicrobial activities against viruses, bacteria and protozoans (Vestal and Jeyaratnam, 2011; Kravets et al., 2016; Wandel et al., 2017; Tretina et al., 2019; Yu et al., 2020). In addition to their established role in host resistance to bacterial and protozoal pathogens, GBPs (e.g., GBP1, GBP2 and GBP5) been have shown to have antiviral activity against human immunodeficiency virus (HIV), Zika virus (ZIKV), hepatitis C virus (HCV), classical swine fever virus (CSFV), dengue virus (DENV), murine norovirus (MNV), influenza virus, hepatitis E virus (HEV), vesicular stomatitis virus (VSV) and encephalomyocarditis virus (EMCV) (Anderson et al., 1999; Nordmann et al., 2012; Pan et al., 2012; Krapp et al., 2016; Li et al., 2016; Yu et al., 2020; Glitscher et al., 2021).

Human GBP1 has been reported to inhibit several flaviviruses such as DENV, HCV and CSFV. In A549 cells, HCV displayed increased replication in the absence of hGBP1 and decreased replication and virus production on over-expression of hGBP1. The activated hGBP1 appeared to exert its inhibitory effect through its large globular GTPase (LG) domain, and binding of HCV-non-structural protein 5B (NS5B) to this domain blocked its GTPase activity and antiviral effect (Itsui et al., 2009; Pandita et al., 2016). A very similar scenario for CSFV was also observed wherein GBP1 suppressed virus replication through its GTPase activity, while CSFV NS5A inhibited GBP1’s antiviral activity *via* blocking its GTPase activity (Li et al., 2016). GBP1 also exhibited an inhibitory effect on DENV infection, through production of antiviral and pro-inflammatory cytokine/chemokines thereby restricting the virus replication (Pan et al., 2012).

Very few studies have suggested the proviral role of GBPs. Murine GBP4 was shown to interact with interferon regulatory factor 7 (IRF7) through its N terminal, thereby disrupting its interaction with TNF receptor-associated factor 6 (TRAF6) and resulting in reduced TRAF6-mediated ubiquitination and transactivation of IRF7 (Hu et al., 2011). In another study GBP7 was shown to promote IAV replication by suppressing innate immune responses to IAV infection *via* inhibition of NF-κB and JAK-STAT signaling pathways along with the production of proinflammatory cytokines and type I and II interferons(IFNs) (Feng et al., 2021).

Here we have examined the role of hGBP1 in JEV infected HeLa cells in an IFNγ unprimed and primed environment. GBP1 was upregulated in JEV infected cells under both conditions. Through immunofluorescence we observed colocalization of GBP1 with NS1 which marks virus replication membranes. GBP1 depletion had no effect on virus replication or cell viability in HeLa cells. However, in an IFNγ primed environment, GBP1 depletion upregulated STAT1 and reduced viral RNA levels and titers suggestive of its proviral role in the context of JEV infection. A similar proviral role of GBP1 was also observed in human neuroblastoma SH-SY5Y cells.

## Materials and Methods

### Cell Lines and Virus

Human epithelial HeLa cell line (CCL-2) and human neuroblastoma SH-SY5Y cell line (HTB-11) were obtained from ATCC VA 20110 USA. Vero and C6/36 cells were obtained from the National Centre for Cell Sciences, NCCS, Pune. JEV strain P20778 (GenBank accession AF080251) was used for all experiments. Vero cells were used for plaque assays to determine virus titres, and C6/36 cell line was used for virus generation. HeLa cells were grown in Dulbecco’s modified Eagle’s medium (DMEM), Vero cells in Eagle’s minimum essential medium (MEM) and C6/36 cells in Leibovitz’s L-15 medium. SH-SH5Y cells were cultured in HiGlutaXL™ DMEM, High Glucose. All cells were supplemented with 10% fetal bovine serum (FBS), 100 µg/ml penicillin/streptomycin and 2 mM L-glutamine.

### Reagents, Antibodies and Primers

Human IFNγ recombinant protein was obtained from eBioscience (14-8311-63). The following primary antibodies were used in the study: GBP1 (ab131255), GAPDH (GTX100118), JEV NS1 (ab41651), STAT1 (p84/p91 sc 346), pSTAT1 (Tyr701-58D6). Polyclonal JEV NS1 and NS3 rabbit antibodies with high specificity and sensitivity were generated in the lab and used for western blot studies. Horseradish peroxidase (HRP)-conjugated secondary antibodies were purchased from Jackson Immunochemicals. ProLong Gold anti-fade reagent with DAPI (P36935) and fluorophore-coupled secondary antibodies were from Invitrogen, Thermo Fisher Scientific. Human ON-TARGETplus- GBP1-smart pool (L-005153-00-0005), ON-TARGETplus control siRNA nontargeting pool (D-001810-10-20), and DharmaFECT 1 transfection reagent (T-2001-02) were purchased from GE Healthcare Dharmacon. The primers (5′-3′) used in the study were as follows: JEV: F-AGAGCACCAAGGGAATGAAATAGT, R-AATAAGTTGTAGTTGGGCACTCTG; JEV TaqMan probe CCACGCCACTCGACCCATAGACTG (5′ end, 6-carboxyfluorescein [FAM]; 3′ end, 6-carb-oxytetramethylrhodamine [TAMRA]); GAPDH:F-TGCACCACCAACTGCTTAGC; R-GGCATGGACTGTGGTCATGAG; GBP1:F- AAGAGAGGACCCTCGCTCTTA; R-ATGCCTTGGTTAGGGGTGAC; GBP2:F- CTATCTGCAATTACGCAGCCT; R-TGTTCTGGCTTCTTGGGATGA; GBP4:F- ATGGGTGAGAGAACTCTTCACG; R-TGCGGTATAGCCCTACAATGG; GBP5:F- CCATGTGCCTCATCGAGAACT; R-ACAGGTTGCGTAATGGCAGAC; GBP6:F- AACCATCTGGCAGGACAGAAT; R-TCACCCTTTTCCACATCGCC; IFNβ:F- CAGGTAGTAGGCGACACTGT; R-TCAATTGCCACAGGAGCTTC.

### Cell Treatment and Virus Assays

Cells were transfected with siRNA for 48 h (NT/GBP1; 25nM), and cell viability, protein, and/or RNA levels of the target gene were checked. At 48 h post-transfection (hpt) cells were infected with virus at 1 MOI, and infection was monitored by harvesting cells for qRT-PCR or western blotting, and supernatant for virus titers. For cell viability assays JEV infection was done at 5 MOI for 48 h. For IFNγ priming, cells were transfected with siRNA for 48 h followed by treatment with 20 ng/ml IFNγ (HeLa cells) and 10 ng/ml (SH-SY5Y cells) for 12 h, followed by JEV infection at the indicated MOI for 24 h.

### RNA Isolation and Quantitative Real-Time PCR

At 24 h post-infection (hpi), the culture supernatant was collected for plaque assays. Total cellular RNA was extracted from cells using RNAiso reagent (Takara Bio) and then isolated using phenol-chloroform method for quantification of transcript levels of various target genes by qRT-PCR. The cDNA was prepared using random hexamers with the GoScript™ Reverse Transcription System (Promega). SyBr mix and TaqMan mix (TaKaRa Bio) were used to set up real-time PCR as described previously (Khasa et al., 2019).

### Western Blot Analysis

Cells were washed with 1 X PBS and then lysed with buffer (1% TritonX-100 in 50 mM Tris-HCl, pH 7.5, 150 mM NaCl, and protease inhibitor cocktail; Sigma-Aldrich, Merck). Protein concentration was estimated using bicinchoninic acid assay (BCA - Pierce 23225). Depending on the molecular weight of the target protein, different percentages of SDS-PAGE were run and equal amount of protein was loaded, which was later transferred to PVDF membrane for immunoblotting. ImageJ software was used to quantitate band intensities. Data are presented as mean values ± standard deviations (SD) obtained from 3 independent experiments.

### Virus Titration by Plaque Assay

Vero cells at 90-95% confluency, were infected with 10-fold serial dilutions of viral supernatant and incubated at 37°C for 1 h with constant rocking. At 1 hpi virus inoculum was removed and cells were washed with 1 X PBS. Overlay agarose plug (1:1, agarose VIII: 2X MEM) was added to the monolayer. Plates were incubated at 37°C until the appearance of well-defined plaques (4-5 days). For titration, the cells were fixed with 3.7% formaldehyde overnight followed by staining with crystal violet solution after removing the agarose plug for visualizing and counting the plaques. The plaque forming units per ml (pfu/ml) were then calculated using the formula: pfu/ml = No. of plaques/(D x V) where D is the dilution factor and V is the volume of virus/well.

### Immunostaining, Fluorescence Microscopy and Image Processing

For the immunofluorescence experiment, cells were seeded on glass coverslips. JEV infection was given at 3 MOI and at 24 hpi, cells were fixed using 2% paraformaldehyde for 15 min followed by permeabilization with 0.3% Tween-20 in 1X PBS for 30 min at RT. Blocking was done using 1% Bovine serum albumin (BSA; Sigma, A7906) in 1X PBS for 1h followed by incubation with primary antibody overnight (JEV-NS1, GBP1). The cells were then washed thrice with 1% BSA for 15 min and stained using specific Alexa Fluor labelled secondary antibodies for 1h at RT. After cells were labelled, mounting was done using ProLong Gold anti-fade reagent with DAPI. Images were acquired on an Olympus FV3000 confocal microscope with 60× (NA 1.4) objective. The co-localization analysis was done using ImageJ software.

### Cytotoxicity Assay

siNT/GBP1 treated cells were infected with JEV at 5 MOI at 48 hpt. Cell viability was measured at 48 hpi using MTT assay (TOX1; Sigma-Aldrich, Merck) as per the product manual.

### Statistical Analysis

Statistical analysis was done using Student’s *t* test. Differences were considered significant at *P* values of <0.05, 0.01, 0.001, and <0.0001, as indicated in the figure legends. Error bars indicate means ± SD (n = 3).

## Results

### GBP1 Expression Is Upregulated During JEV Infection and Its Depletion Does Not Affect JEV Replication in an IFNγ Unprimed Environment

**Figure 1:**
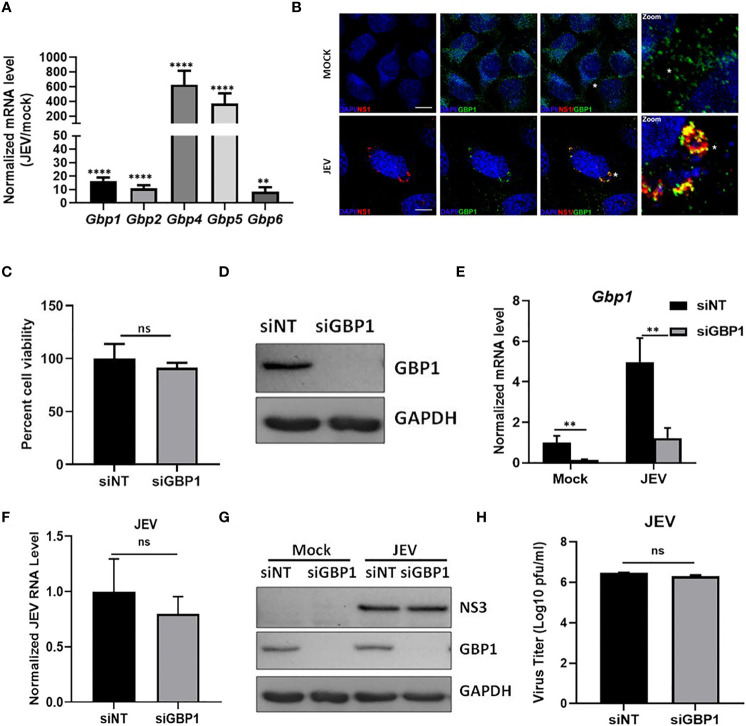
GBP1 depletion does not affect JEV replication in an IFNγ unprimed environment. **(A)** HeLa cells were mock/JEV infected (1 MOI) for 24 h, and mRNA levels of Gbps were quantified by qRT-PCR. Graph shows the relative expression level of gene transcripts in mock/JEV infected samples. **(B)** Mock/JEV-infected (3MOI) HeLa cells were fixed at 24 hpi and immunostained for GBP1 and JEV NS1. Images were acquired on a confocal microscope using a ×60 objective. Scale bar 10µm. **(C)** Western blot showing depletion of GBP1 in siRNA treated HeLa cells at 48 hpt. **(D)** HeLa cells transfected with NT/GBP1 siRNA for 48 h, were mock/JEV infected (1 MOI, 24 h), and GBP1 mRNA levels were determined by qRT-PCR. **(E)** HeLa cells transfected with NT/GBP1 siRNA for 48 h, were either mock/JEV (5 MOI) infected. At 48 hpi, cell viability was determined by MTT assay. Data is shown normalized to NT-siRNA transfected mock treated cells. **(F–H)** HeLa cells transfected with NT/GBP1 siRNA for 48 h, were either mock-or JEV infected (1 MOI, 24 h), and JEV RNA levels were determined by qRT-PCR **(F)**, cell lysates were analyzed by western blotting with JEV-NS3, GBP1 and GAPDH (loading control) antibodies **(G)**, and virus titer was quantitated by plaque assays **(H)**. Western blots are representative of three or more independent experiments. Data presented is mean ± SD of values obtained from 3 independent experiments. Student *t*-test was used to calculate *P* values. **P < 0.01, ****P < 0.0001. ns, Non significant.

Several viruses like HIV, CSFV, VSV, IAV and DENV, have been reported to activate GBPs upon infection (Anderson et al., 1999; Itsui et al., 2009; Nordmann et al., 2012; Pan et al., 2012; Li et al., 2016). We first determined whether the expression of GBPs gets modulated during JEV infection in HeLa cells, and observed significantly enhanced transcript levels of Gbp1, Gbp2, Gbp4, Gbp5 & Gbp6 (**Figure 1A**). We further performed immunostaining experiments and observed GBP1 to be distributed in the perinuclear region in mock infected cells (**Figure 1B**, upper panel). In virus-infected cells GBP1 showed extensive colocalization with the JEV-NS1 protein (**Figure 1B**, lower panel). Our earlier studies have established that JEV-NS1 structures represent virus replication membranes (Sarkar et al., 2021). Pearson’s coefficient of co-localization for GBP1-NS1 in infected HeLa cells was observed to be 0.6, suggesting that GBP1 is recruited to the virus replication complex. To further determine the functional role of GBP1, the endogenous protein was depleted by siRNA treatment and significant knockdown was obtained both at protein and mRNA levels (**Figures 1C, D**). The GBP1-depleted HeLa cells were infected with JEV at 5 MOI, and cell viability was monitored till 48 hpi (**Figure 1E**). A higher MOI was used for measurement of cell viability as JEV infected HeLa cells do not show significant cell death at 1 MOI till 48-72 hpi. GBP1 depletion did not significantly alter cell viability in mock infected cells. JEV infection resulted in ~ 70% cell death in HeLa cells at 24 hpi, and this did not change under GBP1 depletion (**Figure 1E**). The levels of viral RNA, JEV NS protein and titres were also unaffected in GBP1 depleted cells (**Figures 1F–H**). These data suggested that GBP1 does not affect JEV replication in HeLa cells.

### IFNγ Restricts JEV Replication

**Figure 2:**
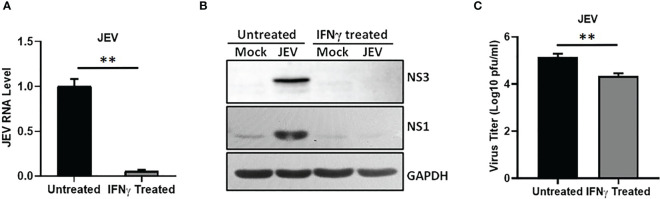
IFNγ restricts JEV replication. HeLa cells were mock or IFNγ (12 ng/ml) treated for 12 h followed by JEV infection at 1 MOI. At 24 hpi, cells were harvested for RNA isolation and cell lysate preparation and supernatant was collected to quantitate virus titer. **(A)** Relative JEV RNA level was analyzed by qRT-PCR, **(B)** Western blots showing the levels of JEV-NS3, JEV-NS1 and GAPDH (loading control). **(C)** Bar graph showing the virus titers determined by plaque assays. Data presented is mean ± SD of values obtained from 3 independent experiments. Student *t*-test was used to calculate *P* values. **P < 0.01.

It is well established that priming with IFNγ provides robust protection against virus infection by activating various ISGs (Rhein et al., 2015). We first assessed the effect of IFNγ priming on JEV replication, and observed a significant inhibition of viral RNA levels (**Figure 2A**), viral protein translation (NS1 and NS3) (**Figure 2B**), and subsequent reduction in infectious virus titers (**Figure 2C**), suggesting an antiviral role of IFNγ for JEV in HeLa cells.

### GBP1 Depletion Reduces JEV Replication and Increases STAT1 and pSTAT1 Levels in an IFNγ Primed Environment

**Figure 3:**
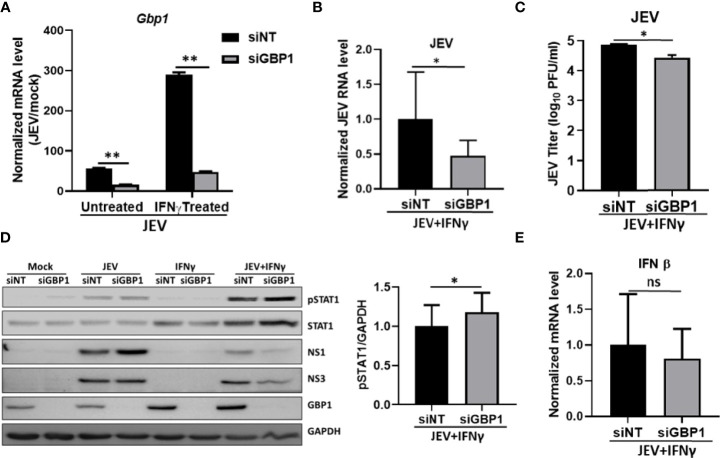
GBP1 depletion reduces JEV replication and increases STAT1 and pSTAT1 levels in an IFNγ primed environment. HeLa cells were transfected with NT/GBP1 siRNA for 48 h, followed by IFNγ treatment for 12 h and then mock or JEV infected at 1 MOI for 24 h. GBP1 mRNA **(A)**, and JEV RNA **(B)** levels were determined by qRT-PCR. **(C)** Virus titer was determined by plaque assays. **(D)** Cell lysates were analysed by western blotting with STAT1, pSTAT1, JEV non-structural proteins (NS1 and NS3), GBP1 and GAPDH (loading control) antibodies. The bar graph shows the levels of pSTAT1 protein in siNT- and siGBP1-treated HeLa cells from three independent experiments. The protein quantitation was done by measuring the band intensities using ImageJ software. **(E)** IFNβ mRNA levels were checked through qRT-PCR. Data presented is mean ± SD of values obtained from 3 independent experiments. Student t-test was used to calculate *P* values. *P < 0.05, **P < 0.01. ns, Non significant.

Studies have shown that IFNγ stimulation leads to enhanced expression of GBPs. We also observed significantly higher levels of GBP1 in JEV infected IFNγ treated cells (**Figures 3A, D**). We next depleted GBP1 using siRNA mediated knockdown, and confirmed it at both transcript and protein levels (**Figures 3A, D**). Interestingly, GBP1 depletion further reduced JEV RNA, protein levels and titers in these IFNγ primed HeLa cells (**Figures 3B–D**). We next examined the effect of GBP1 knockdown on JEV-induced activation of JAK-STAT signaling and expression of IFNβ. We observed upregulated levels of STAT1 and its enhanced phosphorylation, but no significant change was seen on IFNβ levels upon GBP1 depletion (**Figures 3D, E**). These data suggest that GBP1 downregulates JAK-STAT signaling in IFNγ primed JEV infected HeLa cells and thereby supports virus replication.

### GBP1 Depletion Reduces JEV Replication in an IFNγ Primed Environment in SH-SY5Y Cells

**Figure 4:**
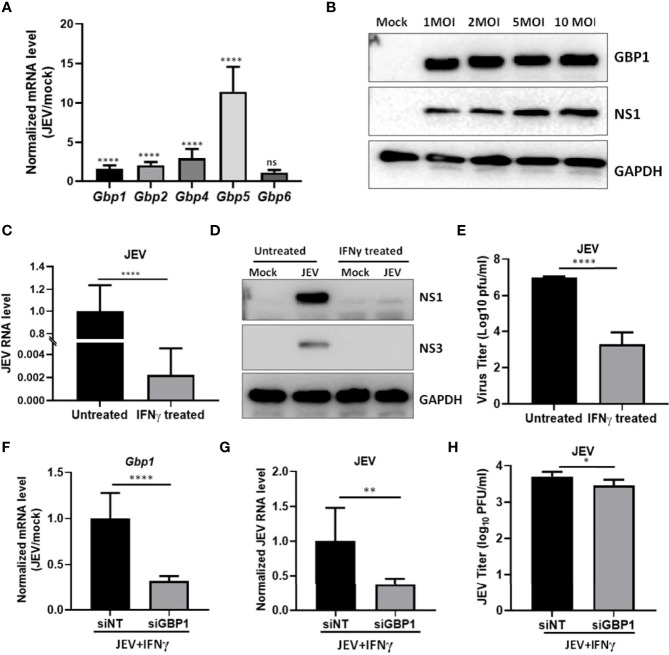
GBP1 expression is upregulated during JEV infection and its depletion reduces JEV replication in an IFNγ primed environment in SH-SY5Y cells. **(A)** SH-SY5Y cells were mock or JEV infected at 1 MOI for 24 h, and mRNA levels of Gbps were quantified by qRT-PCR. Graph shows the relative expression level of gene transcripts in mock and JEV infected samples. **(B)** SH-SY5Y cells were mock or JEV infected at 1, 2, 5 and 10 MOI for 24 h, cell lysates were analyzed by western blotting with JEV non-structural protein (NS1), GBP1 and GAPDH (loading control) antibodies. **(C–E)** SH-SY5Y cells were mock or IFNγ (10 ng/ml) treated for 12 h followed by JEV infection at 1 MOI. At 24 hpi, cells were harvested for RNA isolation or cell lysate preparation, and supernatant was collected to quantitate virus titer. **(C)** JEV RNA level was analyzed by qRT-PCR, **(D)** Western blots showing the levels of JEV-NS3, JEV-NS1 and GAPDH (loading control), **(E)** Bar graph showing the virus titer determined by plaque assays. **(F–H)** SH-SY5Y cells were transfected with NT/GBP1 siRNA for 48 h, followed by IFNγ treatment for 12 h and then mock or JEV infected at 1 MOI. At 24 hpi total RNA was isolated and GBP1 mRNA **(F)**, and JEV RNA, **(G)** levels were determined by qRT-PCR. **(H)** Virus titer was determined by plaque assays. Data presented is mean ± SD of values obtained from 3 independent experiments. Student t-test was used to calculate P values. *P < 0.05, **P < 0.01, ****P < 0.0001. ns, Non significant.

We further validated our results in JEV infected human neuroblastoma SH-SY5Y cells, where we observed significantly enhanced transcript levels of Gbp1, Gbp2, Gbp4 & Gbp5 (**Figure 4A**). We also found the upregulation of GBP1 protein upon virus infection (**Figure 4B**). IFN**γ** priming resulted in a significant inhibition of viral RNA levels (**Figure 4C**), viral protein translation (NS1 and NS3) (**Figure 4D**), and infectious virus titers (**Figure 4E**), validating an antiviral role of IFN**γ** in SH-SY5Y cells. Next, GBP1 was depleted by siRNA treatment in the IFN**γ** primed environment (**Figure 4F**), where a further significant reduction in JEV RNA levels and titers was observed (**Figures 4G, H**), similar to our observations in HeLa cells.

## Discussion

GBPs are a family of IFN-inducible GTPases belonging to the dynamin superfamily with several common ISG characteristics. GBPs are expressed in response to Type I & II IFN, and other inflammatory cytokines like IL-1α, IL-1β, and TNF-α. IFNγ has been shown to induce hGBPs 1 to 5 in cultured endothelial cells. Studies have established that GBPs are crucial for inflammasome activation and antimicrobial activities (Vestal and Jeyaratnam, 2011; Man et al., 2017; Ngo and Man, 2017).

GBP1 has been shown to exert antiviral effects against several viruses including VSV, EMCV, HSV-1 and flaviviruses such as HCV and DENV (Anderson et al., 1999; Nordmann et al., 2012; Pan et al., 2012; Krapp et al., 2016; Li et al., 2016; Yu et al., 2020; Glitscher et al., 2021). Mechanistically GBPs and their associated family members have been shown to target and lyse the pathogen-containing vacuole membranes and destroy the residential habitat of vacuolar protozoan and bacterial pathogens (Ngo and Man, 2017; Praefcke, 2018). Several reports have suggested that IFN priming and induction of GBPs is crucial for caspase-4 (murine caspase-11) dependent pyroptosis and destruction of the bacterial replication niche (Meunier et al., 2014; Pilla et al., 2014; Santos et al., 2020; Wandel et al., 2020). A few recent studies have shown that GBPs can also exert a proviral influence highlighting that their role is likely to be highly complex and divergent depending on the virus and host cell (Hu et al., 2011; Feng et al., 2021).

Robust activation of GBPs in response to JEV infection at both the transcript and protein levels has been shown in our previous studies on human monocyte derived dendritic cells and mouse embryonic fibroblasts (Chauhan et al., 2021; Sharma et al., 2021a). Here, we found that GBP1 expression was markedly induced in human epithelial HeLa cells upon JEV infection, and interestingly GBP1 strongly colocalized with JEV-NS1 protein that marks the virus replication complex. We speculated that GBP1 recruitment to the virus replication complex could be an antiviral host defense mechanism similar to what has been described for bacterial pathogens (Ngo and Man, 2017; Praefcke, 2018). However, GBP1 depletion did not affect viability of virus infected cells and had no effect on the virus replication cycle. Since GBP’s are strongly induced through IFNγ, we next tested the function of GBP1 in an IFNγ primed environment. Here, surprisingly, GBP1 knockdown reduced viral RNA and protein levels and titres, indicative of its proviral role. This appeared to be mediated through negative modulation of the innate immune response, as enhanced activation and phosphorylation of STAT1 was observed in GBP1 depleted cells. These data suggest that GBP1 facilitates JEV replication by suppressing the innate immune response *via* the JAK-STAT signaling pathway but has no effect on IFNβ induction. The proviral role of GBP1 in the IFNγ primed environment was also validated in neuronal SH-SY5Y cells. It is also possible that other GBPs or host factors, might play a concerted role with GBP1 during infection. Our observation also highlights that the role of GBPs in the context of virus infection is likely to be highly complex.

## Data Availability Statement

The raw data supporting the conclusions of this article will be made available by the authors, without undue reservation.

## Author Contributions

SC planned and performed all the experiments, analyzed the data and wrote the manuscript. KS contributed to study design, data analysis and reviewed the manuscript. MK supervised the research and finalized the manuscript. All authors contributed to the article and approved the submitted version.

## Funding

This work was supported by a grant from DBT BT/PR27875/Med/29/1302/2018, and from DBT intra-mural funds to RCB. SC was supported by DBT Ramachandran fellowship.

## Conflict of Interest

The authors declare that the research was conducted in the absence of any commercial or financial relationships that could be construed as a potential conflict of interest.

## Publisher’s Note

All claims expressed in this article are solely those of the authors and do not necessarily represent those of their affiliated organizations, or those of the publisher, the editors and the reviewers. Any product that may be evaluated in this article, or claim that may be made by its manufacturer, is not guaranteed or endorsed by the publisher.
